# The efficacy of prophylactic metoclopramide in preventing nausea and vomiting in patients with acute pain treated with intravenous tramadol: a randomized double-blinded, placebo-controlled trial

**DOI:** 10.1186/s13104-023-06395-y

**Published:** 2023-07-27

**Authors:** Haruethai Sriwiset, Thanin Lokeskrawee, Jayanton Patumanond, Pakpoom Wongyikul, Phichayut Phinyo

**Affiliations:** 1 Department of Emergency Medicine, Lampang Hospital, Muang District, Lampang, 52000 Thailand; 2 Center for Clinical Epidemiology and Clinical Statistics, Faculty of Medicine, Chiang Mai University, Chiang Mai, 50200 Thailand; 3 Department of Family Medicine, Center for Clinical Epidemiology and Clinical Statistics, Musculoskeletal Science and Translational Research, Faculty of Medicine, Chiang Mai University, Chiang Mai, 50200 Thailand

**Keywords:** Analgesics opioid, Opioid induce nausea and vomiting, Randomized controlled trial, Tramadol, Metoclopramide

## Abstract

**Objective:**

To examine the clinical efficacy of prophylactic metoclopramide in reducing the incidence of nausea and vomiting in emergency department (ED) patients with acute pain who were treated with intravenous tramadol.

**Results:**

We conducted a single-center randomized, double-blinded, placebo-controlled trial. A total of 99 ED patients presented with acute pain were recruited. Sixty-four patients were randomized, 31 patients in the treatment arm and 33 in the control arm. Overall, there were no significant differences in baseline characteristics between treatment arm and control arm. Only one patient within each arm reported having nausea symptom. No patients reported vomiting episode. There was no statistically significant difference in the proportion of patients with nausea or vomiting symptoms between the two groups (3.2% in the treatment arm vs. 3.0% in the control arm, p = 1.000). The administration of prophylactic metoclopramide may not provide additional benefit in reducing the occurrence of nausea and/or vomiting episode in ED patients with acute pain treated with intravenous tramadol.

*Trial registration* Randomized clinical trial TCTR20220525001; registration date: 21 October 2021. Retrospectively registered.

## Introduction

Acute pain is a common chief complaint in the emergency department (ED) [1, 2]. Intravenous opioid is generally prescribed by ED physicians to alleviate the patient’s distress and interrupt the pain cycle [3]. However, apart from its potency, previous studies had reported several side effects of strong opioids, including sedation, dizziness, nausea, vomiting, and respiratory depression [4, 5]. Moreover, as there was a high potential for addiction after chronic use, strong opioid was classified as a controlled substance in many countries [6]. In Thailand, tramadol, a weaker subclass of opioid, has become a more attractive drug of choice for initial control of moderate to severe pain [7]. Unlike other strong opioids, Tramadol has no clinically relevant effects on respiratory or cardiovascular parameters, and also has a low potential for drug abuse at recommended doses [8, 9]. However, as tramadol was a central acting agent, the incidence of gastrointestinal adverse events, such as opioid-induced nausea and vomiting (OINV), is still common, especially when the drug was delivered in parenteral form [10, 11].

Strategies were proposed to reduce the side effects of intravenous opioid [3]. One of which is the use of prophylactic metoclopramide [3]. Although it was long presumed that routine administration of prophylactic metoclopramide might be beneficial, the clinical evidence for its efficacy in reducing OINV in acute pain setting is still unclear [12, 13]. Recently, a Cochrane review including three randomized trials concluded that prophylactic metoclopramide failed to provide meaningful benefit in reducing the risk of nausea, vomiting, and the need for rescue medication. However, the overall body of evidence was still of low certainty [13]. We aimed to conduct a randomized placebo-controlled trials to examine the clinical efficacy of prophylactic metoclopramide in reducing the incidence of nausea and vomiting in ED patients with acute pain and were treated with intravenous tramadol.

## Method

### Study design and setting

This study was a randomized, double-blinded, placebo-controlled trial conducted in the Emergency department of Lampang Hospital, Thailand from October 2021 to March 2022. The study protocol was registered in Thai Clinical Trials Registry (TCTR) [TCTR20220525001]. The Institutional Review Board of Lampang Hospital approved the study protocol (CERT NO. 103/64).

### Participants and data collection

We include patients who presented to the ED during the study period who fulfilled the following criteria: (1) aged more than or equal to 18 years, and (2) presented with any type of acute painful conditions, including headache, musculoskeletal pain, renal colic, gallbladder colic, with moderate to severe intensity (pain scale ≥ 5 in Numerical Rating Scale (NRS)). Patients with one of the following conditions were excluded from the study: pregnancy or lactation, contraindicated to tramadol or metoclopramide, had nausea or vomiting symptoms upon study inclusion, or at the time of randomization, patient who were at high risk for extrapyramidal side effects, patients who were treated with drugs other than intravenous Tramadol, patients who could not provide informed consent, and patients with previous antiemetic use.

We collected baseline data on age, sex, underlying disease, location of pain, and severity of pain recorded in NRS.

### Randomization of participants

All included patients were enrolled and randomized into one of the two groups in a 1:1 ratio, either prophylactic metoclopramide plus intravenous tramadol (treatment arm) or placebo plus intravenous tramadol (control arm), using a computerized blocked randomization generated by an independent research assistant. The randomization sequences were also stratified based on age groups and sex. Allocation concealment was performed using sequentially-numbered opaque sealed envelopes (SNOSE). The sealed envelopes were opened just after the patients were enrolled into the study and all baseline data were collected. The study interventions were provided immediately after randomization.

### Interventions and blinding

Patients randomized into the treatment arm received 50 mg intravenous tramadol and 10 mg intravenous metoclopramide. In contrast, patients in the control arm were administered with 50 mg intravenous tramadol and a matching placebo of intravenous metoclopramide, consisting of 10 ml of normal saline solution (NSS). The treatment was prepared and administered by ED nurses who were not involved in the outcome assessment. Patients, attending physicians, data collectors, and outcome assessors were all blinded to the study assignments.

For both arms, an intravenous tramadol (50 mg/1 mL) was diluted with 19 mL of NSS, summed up to a total volume of 20 mL, and was slowly pushed within 2 min. After intravenous tramadol was delivered, a 10 mL of NSS was used for flushing the infusion line. Then, either an intravenous metoclopramide (10 mg/2 mL) diluted with 8 mL of NSS or a matching placebo containing 10 mL of NSS were administered within the next minute for the treatment and control arm, respectively.

### Outcome measurement and follow-up

The primary outcome was the occurrence of nausea and/or vomiting within 2 h after randomization [13]. Nausea was defined as a subjectively unpleasant sensation, usually described as a conscious awareness of the need to vomit [14], whereas vomiting was an objective event that results in a forceful evacuation of gastric contents from the stomach out of the mouth [14]. The secondary outcome was the severity of the nausea which was categorized into four levels: none, mild (nausea symptom without the need for rescue therapy), moderate (nausea and vomiting symptom requiring rescue therapy with single anti-emetic agent), and severe (nausea and vomiting symptoms with more than one anti-emetic agents prescribed).

The primary outcome was assessed by independent health care providers who were blinded to the treatment assignments. The outcome assessors evaluated the presence/absence of nausea or vomiting symptom, the severity, the need for rescue therapy or anti-emetic agent, and the pain NRS every 30 min until patient disposition or 2 h after randomization, which ever were reached first.

### Statistical analysis

According to our routine observations, the incidence of nausea and vomiting in ED patients with acute pain treated with intravenous tramadol was approximately 30%. We assumed that administration of intravenous metoclopramide would result in a reduction of nausea and vomiting incidence by 25 percentage points compared to placebo. To achieve statistical power of 80% at 5% one-sided significance level, with an anticipated 10% drop-out rate, a total of 64 patients was required.

Categorical data were described with frequency and percentage. Mean and standard deviation (SD) for Normally-distributed numerical data, and median and interquartile range (IQR) for non-Normally distributed numerical data. The normality of data was justified based on histogram and Shapiro–Wilk test. The incidence of nausea and/or vomiting between the two treatment arms were compared using Fisher’s exact probability test (one-sided). The analysis was performed using the intention-to-treat principle where all patients were included in the analysis and were analyzed according to which group they were initially assigned. P values less than 0.05 were considered statistically significant differences. All statistical analyses were conducted using Stata 17 (StataCorp, College Station, TX, USA).

## Result

**Fig. 1:**
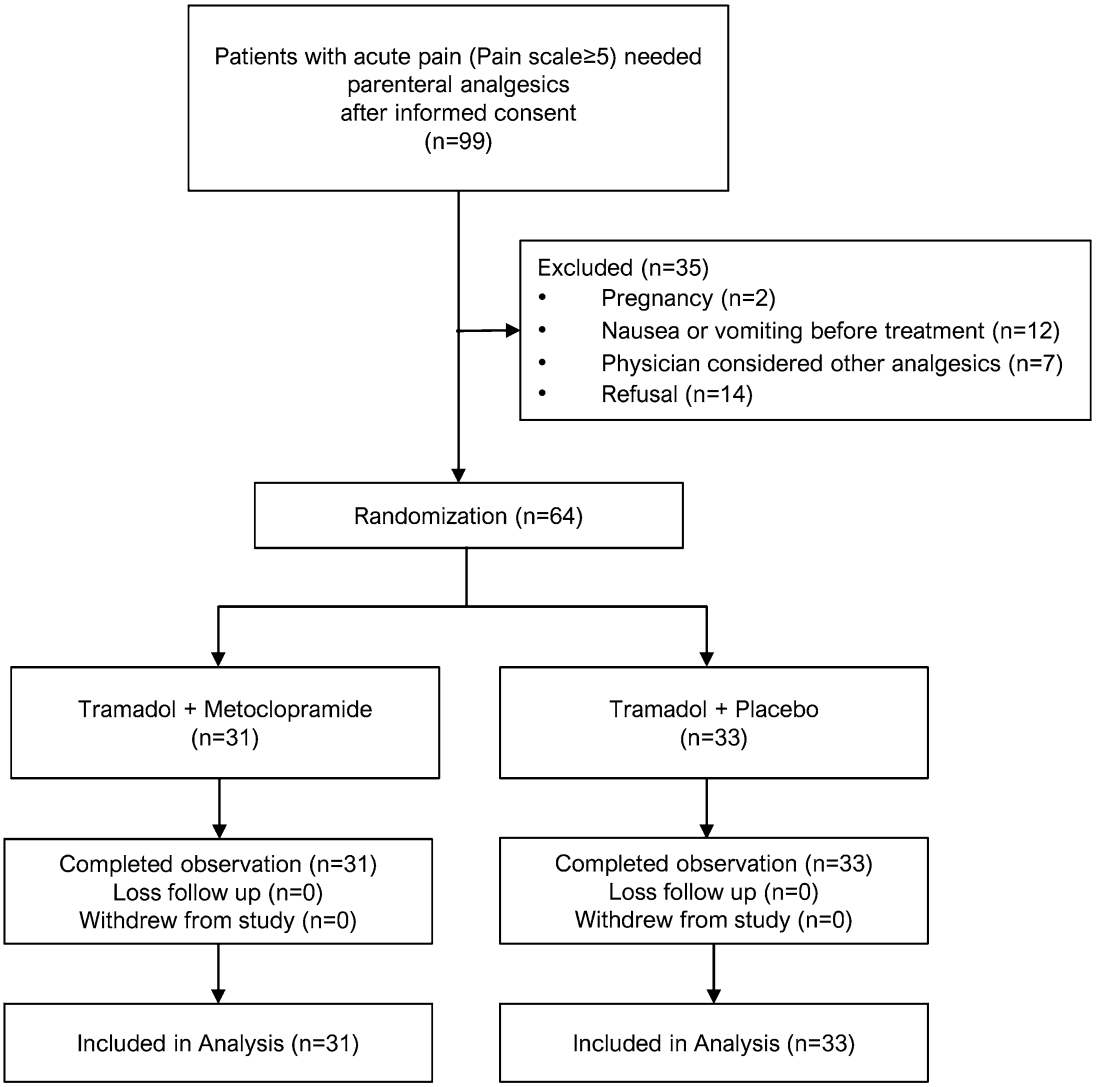
CONSORT flow diagram

A total of 99 ED patients presented with acute pain were recruited. Sixty-four patients were randomized, 31 patients in the treatment arm and 33 in the control arm (Fig. 1). In term of demographic, means age were similar in both groups at approximately 55 years and Female was a dominant proportion. Location of pain, Extremities and Urinary system were equally the most leading cause of pain in both groups. Overall, there were no significant differences in baseline characteristics between treatment arm and control arm (Table 1).

**Table 1:** Baseline clinical characteristics of the included patients

Characteristic	Tramadol plus Metoclopramide (n = 31) n (%)	Tramadol plus Placebo (n = 33) n (%)
*Gender*		
Male	12 (38.7)	15 (45.5)
Female	19 (61.3)	18 (54.5)
Age (years), mean ± SD	55.2 ± 16.4	54.4 ± 17.2
*Underlying disease*		
Central nervous system	5 (16.1)	3 (9.1)
Gastrointestinal	0 (0)	0 (0)
Psychiatric	0 (0)	0 (0)
*Location of pain*		
Head	4 (12.9)	2 (6.1)
Neck	0 (0)	0 (0)
Chest wall	4 (12.9)	8 (24.2)
Abdominal wall	1 (3.2)	0 (0)
Back	5 (16.1)	6 (18.2)
Extremities	8 (25.8)	8 (24.2)
Gastrointestinal tract	0 (0)	1 (3.0)
Urinary system	8 (25.8)	8 (24.2)
Reproductive organ	0 (0)	0 (0)
Other	1 (3.2)	0 (0)
*Pain scale (Initial), median [IQR]*	7 [5, 8]	7 [6, 10]
*Pain scale (Disposition), median [IQR]*	2 [0, 3]	3 [0, 4]

For the primary outcome, only one patient within each arm reported having nausea symptom. No patients reported vomiting episode. There was no statistically significant difference in the proportion of patients with nausea or vomiting symptoms between the two groups (3.2% in the treatment arm vs. 3.0% in the control arm, p = 1.000) (Table 2). No adverse event of intravenous metoclopramide was reported in this study.

**Table 2:** Incidence of nausea and vomiting and number of patients experience nausea for each severity between the two treatment groups

	Tramadol plus Metoclopramide (n = 31) n (%)	Tramadol plus Placebo (n = 33) n (%)	P value
*Incidence of nausea/vomiting*			
Nausea	1 (3.2)	1 (3.0)	1.000
Vomiting	0 (0)	0 (0)	NA
*Severity of nausea/vomiting*			
None	0 (0)	0 (0)	1.000
Mild	1 (3.2)	1 (3.0)	
Moderate	0 (0)	0 (0)	
Severe	0 (0)	0 (0)	

*NA* not applicable

## Discussion

In this randomized placebo-controlled trial, we have found that administration of prophylactic metoclopramide may not provide additional benefit in reducing the occurrence of nausea and/or vomiting episode in ED patients with acute pain treated with intravenous tramadol.

The results of our study were, however, in contrast to that of Kim et al. in 2019 [15]. In their study, a total of 191 trauma patients whose pain were treated with intravenous tramadol were randomized into two study groups, 96 assigned to prophylactic metoclopramide and another 95 to placebo. It was found that the administration of prophylactic metoclopramide resulted in a significant reduction in OINV (0% in prophylactic metoclopramide group vs. 5.3% in placebo group). The discrepancy in the findings and conclusions between the two studies could be explained by the different in their designs. In our study, we included ED patients with a wider range of pain spectrum, whereas only traumatic patients with musculoskeletal pain were eligible in Kim’s study [15]. This difference in the patient domain may affect both the baseline risk of OINV and the treatment effect of prophylactic metoclopramide in reducing OINV [16, 17]. Another potential explanation was how intravenous tramadol was administered [18]. Previous evidence has shown that higher dosages and higher concentrations of opioids could increase the possibility of adverse events [19, 20]. According to our study protocol, 50 mg of intravenous tramadol was diluted with NSS and slowly infused over a 2-min interval. Diluting the concentration of opioid agents is a common preventive strategy for OINV [21], which might explain the low incidence of nausea and vomiting in our patient cohort. However, Kim's study did not state how tramadol was delivered [15]. Thus, it is difficult to compare the results between studies. If no preventive strategy was used to prevent OINV, this might explain the higher incidence of the outcome in Kim’s control group compared to ours. However, if a certain preventive strategy was used, the higher incidence of OINV would likely be attributed to differences in patient domains and baseline OINV risk.

This study was the first randomized, placebo-controlled trial to be conducted in Thai patients who presented to ED with acute pain symptoms and treated with intravenous tramadol. Although we could not provide firm conclusions on the benefits of prescribing prophylactic metoclopramide, our study still serves as an important contradicting evidence that addresses this clinically relevant question in a wider spectrum of patient population.

### Limitations

The results of our study should be interpreted in light of limitations. First, although the required sample size was achieved, the incidence of nausea and vomiting symptoms in our sample was unexpectedly low, and the anticipated effect of prophylactic metoclopramide over placebo was not observed. It is obvious that our predetermined study size was not sufficiently powered. This low incidence issue was also observed in previous studies in which a higher potency of opioid was used [22–24] and no clinically important difference was identified [13, 22–24]. It might be safe to infer that prophylactic intravenous metoclopramide may not be beneficial in a low incidence setting or to a patient domain with a low risk of OINV. Further studies targeting patients with higher risks of OINV, such as those who have a previous history of OINV, women, or young age [25] might be able to demonstrate the clinical value of prophylactic metoclopramide or other potential agents, such as ondansetron. Second, the etiologies of nausea and vomiting symptoms within 2 h after randomization were difficult to justify whether they were the consequences of intravenous tramadol, or the accompanied symptoms of the presenting pain conditions. However, as patients who reported nausea and vomiting symptoms before the point of randomization were excluded, it was more likely that the events occurring after randomization were related to tramadol. Finally, this study was conducted in an ED of a single tertiary care center in Northern Thailand. The results of our study might only be generalizable to clinical settings with similar backgrounds to ours.

In conclusion, the clinical benefit of prescribing prophylactic metoclopramide to reduce the occurrence of nausea and vomiting symptoms in ED patients with acute pain treated with intravenous tramadol was not identified in this trial. Therefore, prescribing prophylactic metoclopramide to reduce the occurrence of OINV to all ED patients may not be necessary in settings with low OINV incidence, such as those where preventive administration strategies have already been implemented. Further studies should focus on the potential efficacy of prophylactic antiemetics in patient domains with a higher baseline OINV risk.

## Data Availability

The datasets used and/or analysed during the current study available from the corresponding author on reasonable request.
